# Adaptive Behavior and Development of Infants and Toddlers with Williams Syndrome

**DOI:** 10.3389/fpsyg.2016.00598

**Published:** 2016-04-28

**Authors:** Rebecca M. Kirchner, Marilee A. Martens, Rebecca R. Andridge

**Affiliations:** ^1^Department of Neuroscience, The Ohio State University, ColumbusOH, USA; ^2^Department of Psychology, The Ohio State University, ColumbusOH, USA; ^3^Department of Psychology, The Ohio State University at Newark, NewarkOH, USA; ^4^Nisonger Center, The Ohio State University, ColumbusOH, USA; ^5^Department of Biostatistics, The Ohio State University, ColumbusOH, USA

**Keywords:** Williams syndrome, adaptive behavior, developmental disability, development, infants and toddlers

## Abstract

Williams syndrome (WS) is a neurodevelopmental disorder that causes deficits in adaptive behavior, difficulties eating and sleeping, cognitive delays, and delayed development. Although researchers have conducted characterizations of children and adults with WS, less is known about young children with this disorder. This study characterizes the developmental and adaptive behavior features of 16 infants and toddlers with WS aged 3 months – 5 years. Data for this project was obtained from 2007 to 2014, and includes parent report data and standardized developmental testing. Thirty-one percent (31.3%) of parents reported that their infant/toddler with WS had sleeping problems and 58.3% reported feeding difficulties. Levels of adaptive behavior were in the Mildly Delayed range as measured by the Adaptive Behavior Assessment System, Second Edition. Self-care skills such as feeding or dressing oneself were significantly weaker than skills needed to function in the community, such as recognizing his/her home or throwing away trash. The difficulty with self-care skills is hypothesized to be related to the reported difficulties with eating and sleeping. Motor skills were significantly lower than both cognitive and language skills on the Bayley Scales of Infant and Toddler Development, Third Edition. The current study highlights the need for early intervention in these young children across all areas of development, particularly in self-care skills.

## Introduction

Williams syndrome (WS) is a neurodevelopmental disorder caused by a deletion of approximately 26–28 genes on chromosome 7 (7q11.23; Peoples et al., 2000). This rare syndrome affects 1 in 7,500 to 1 in 20,000 individuals (Wang et al., 1997; Strømme et al., 2002) and is genetically confirmed via florescence *in situ* hybridization or microarray analysis (Lowery et al., 1995). Distinctive physical characteristics are evident (Morris and Mervis, 1999), and many individuals with WS experience hyperacusis, defined as an oversensitivity to sound (Gothelf et al., 2006). Research has demonstrated that this oversensitivity is the most severe during early childhood, and declines as the child ages (Gothelf et al., 2006). Cardiovascular abnormalities are another common characteristic of individuals with WS, and occur in 82% of individuals (Collins et al., 2010). The most commonly occurring cardiac abnormalities are supravalvular aortic stenosis and peripheral pulmonary stenosis, occurring in 45% and 37% of individuals with WS, respectively (Collins et al., 2010). Individuals with WS are also smaller than average, with 70% of a 42-person sample having a birth length and weight less than the 10th percentile on a normal growth curve (Morris et al., 1988; Martin et al., 2007). Children with WS also have a unique behavior profile, which includes difficulties with eating and sleeping (Davies et al., 1998; Annaz et al., 2011). Axelsson et al. (2013) found that infants and toddlers with WS had significantly reduced and disrupted sleep, as well as higher emotionality and attention problems, as compared to typically developing young children. Failure to thrive has also been reported in infants with WS, as well as feeding difficulties, reflux, and constipation (Morris, 2010).

Cognitive delays are also common in individuals with WS, with 75% of young children demonstrating IQ and adaptive behavior scores consistent with a developmental delay, and the remaining 25% exhibiting learning disabilities (Mervis and Klein-Tasman, 2000). A study by Mervis and John (2010) determined the average General Conceptual Ability score on the Differential Ability Scales-II (DAS-II; Elliott, 2007) to be 64.56, with a majority of children performing significantly better on either the Verbal or Nonverbal reasoning cluster than on the Spatial cluster (Mervis and John, 2010). The DAS-II is an assessment designed to examine children’s cognitive strengths and weaknesses, and it is important to note that the General Conceptual Ability scores have an age-based standard score of 100 (Elliott, 2007). As the aforementioned study suggests, individuals with WS typically display relative strengths in concrete language and nonverbal reasoning, and a severe weakness in visuospatial construction (Mervis and John, 2010).

While a fair amount of research has been conducted on children, adolescents, and adults with WS, less developmental research has been focused on the infant/toddler age range. Using the Bayley Scales of Infant Development (Bayley, 1969), Mervis and Bertrand (1997) found that infants and toddlers overall correctly answered more verbal questions than non-verbal items, and demonstrated extreme difficulties with tasks relating to visuospatial construction. In the area of cognition, they found nearly all infants and young children with WS displayed a developmental delay.

The American Association on Intellectual and Developmental Disabilities (AAIDD) defines adaptive behavior as the collection of conceptual, social and practical skills that individuals need to function in their daily lives (Schalock et al., 2010). According to parental reports using the Vineland Adaptive Behavior Scales, Interview Edition (VABS; Sparrow et al., 1984), children with WS demonstrate deficits in adaptive behavior (Greer et al., 1997). Greer and colleagues found that 40% of children and adolescents with WS had overall Adaptive Composite Scores (*M* = 100, *SD* = 15) in the Moderately Deficient range (40–55), 33% scored within the Mildly Deficient range, (55–70) and 27% scored in the Moderately Low range (70–85). Communication and socialization skills appeared more developed than daily living skills, and no gender differences were found (Greer et al., 1997). In regard to overall strengths and weaknesses within the adaptive behavior domains, heterogeneity is evident, with no one overall area identified as a significant strength or weakness (Brawn and Porter, 2014).

Another, more specific study was conducted concerning the adaptive behavior skills of 41 four through 8-year-old children with WS (Mervis et al., 2001). The results, gathered from the VABS, showed strengths in socialization skills, as well as a strength in communication skills in comparison to daily living skills. The motor skills of the 4 and 5-year-old children were characterized, and the results showed a significant weakness in motor skills relative to the other areas. A comparison of adaptive standard scores and chronological age suggested that as young children with WS increase in age, their position relative to their same age peers remains stable. In other words, as children with WS grow older, their adaptive behavior skills are following a delayed yet parallel trajectory compared to their same aged peers.

A recent study looked at the adaptive behavior profile of children with WS under the age of 5 years using the VABS (Hahn et al., 2014). Within a cohort of 18 individuals with WS (mean age of 47.61 months), mean scores were assessed to look for the adaptive behavior profile of young children with WS. In this sample, pairwise comparisons indicated significantly higher scores on the Communication domain (*M* = 74.44) in comparison to the Daily Living domain (*M* = 60.11). In addition, the Socialization domain (*M* = 73.83) was found to have a significantly higher mean score than the Daily Living domain and the Motor domain (*M* = 61.28). The Communication domain was found to have a significantly higher mean score in comparison to the Motor domain as well.

As the literature demonstrates, there has been a considerable amount of research investigating the developmental and adaptive behavior characteristics of individuals with WS, but less is known about the subpopulation of infants and toddlers. This is the first study to assess the adaptive behavior of infants and toddlers with WS using the Adaptive Behavior Assessment System, Second Edition (ABAS-2; Harrison and Oakland, 2003). The domains of the ABAS-2 (Conceptual, Social, and Practical) follow the aforementioned AAIDD guidelines for measuring adaptive behavior, in contrast to the Communication, Daily Living Skills, and Socialization domains utilized in the VABS, which has been used in previous studies. This research characterizes the overall developmental and adaptive behavior phenotype of infants and toddlers with WS, and examines whether there are any strengths or weaknesses in the profiles of these young children that may point to specific areas of needed intervention.

## Materials and Methods

### Participants

The participants in this study were 16 individuals (11 females, 5 males) aged 3 months to 65 months (*M* = 28.7 months, *SD* = 19.3 months) who attended a WS Clinic in the United States. In order to be eligible for this study, participants had to be under the age of 6 years when they were first seen by the clinic. Only data from the child’s first clinic visit was utilized in this study. Five additional individuals within this age range were excluded from the study due to absence of the correct intake form (*n* = 3) or because the child also met criteria for an Autism Spectrum Disorder (*n* = 2). Data for this study comes from paper report questionnaires completed by the child’s primary caregiver, as well standardized evaluations given while the individuals were at the Clinic.

This study was carried out in accordance with the recommendations of Nationwide Children’s Hospital Institutional Review Board, with written informed consent from the primary caregivers of all subjects. All primary caregivers gave written informed consent in accordance with the Declaration of Helsinki.

### Materials

#### Williams Syndrome Intake Form

The WS Intake Form (see **Supplementary Figure S1**) was given to the parents or guardians of each child upon their first visit to the WS Clinic. Parents or guardians of the child completed all sections to the best of their knowledge. The information asked on the form includes the following: basic demographic information, history of feeding difficulties, persistent and/or past health problems, sleep difficulties, and questions relating to hyperacusis.

#### Bayley Scales of Infant and Toddler Development, Third Edition

The Bayley Scales of Infant and Toddler Development, Third Edition (Bayley-III; Bayley, 2006), a clinician administered assessment, evaluates a child in three areas: Cognitive, Language (consisting of Receptive and Expressive Language subtests), and Motor (consisting of Fine and Gross Motor subtests). The Cognitive, Language, and Motor scales have a mean of 100 and a standard deviation of 15, while the subtests have a mean of 10 and a standard deviation of 3. The Bayley-III normative data consisted of 1700 infants, 10% of which contained children with specific clinical diagnoses such as developmental delay (Bayley, 2006). The Bayley-III has been found to have both acceptable reliability and validity in both normative and clinical groups.

#### Adaptive Behavior Assessment System, Second Edition

The Adaptive Behavior Assessment System, Second Edition, completed by the child’s primary caregiver, was used to assess the child’s personal and social skills necessary for daily living. The ABAS-2 measures adaptive behavior in three domains, Conceptual (Communication and Pre-Academic skills), Social (Interpersonal and Social Competence skills), and Practical (Daily Living Skills), and provides a General Adaptive Composite score. The domains, along with the General Adaptive Composite, have a mean score of 100, and a standard deviation of 15. The three domains are comprised of nine sections, or skill areas, which have a mean of 10 and a standard deviation of 3. Additionally, there is a Motor or Work skill area (depending on the age of the child), which does not make up one of the three domains. It is important to note that when the child is under the age of one year, three of the skill areas, Community Use (using appropriate behavior in the community), Functional Pre-Academics (beginning academic skills), and Home Living (taking care of basic home needs), are not included in the computation of the adaptive behavior scores. Ratings are based on a 4-point Likert-type scale ranging from 0 to 3; with 0 meaning that the child is not able to perform the listed behavior, and 3 meaning that the child is always or almost always able to perform the behavior when needed. The ABAS-2 has been found to have a high degree of internal consistency, and demonstrates high concurrent validity with the VABS (Harrison and Oakland, 2003).

### Procedures

All data used in this study was retrospective, taken from the aforementioned assessments and forms. Although the intake form contained many questions, for the purpose of this study, only the following information was used: birthdate, sleep and feeding difficulties, use of a special diet, and evidence of hyperacusis (heightened sensitivity to certain sounds). Certain variables do not have full sample participation for reasons such as: parents skipping the question, records not being faxed over from other hospitals, or incomplete assessments.

### Statistical Methods

Normality tests for continuous measures indicated that parametric tests were appropriate for all analyses in this data set. Therefore, repeated measures analysis of variance and paired sample *t*-tests were used to test for within-subject differences across areas of the Bayley-III and ABAS-2. In addition, Pearson correlations were used to quantify relationships between mental age and chronological age on the ABAS-2 skill areas. All analyses were performed in SPSS version 22.

## Results

### Developmental/Cognitive

The parent-reported intake forms indicated issues with sleeping and eating, as anticipated. Thirty-one percent (31.3%) of parents reported that their infant/toddler had sleeping problems, 58.3% reported feeding difficulties, and 42.9% reported that their infant/toddler needed a special diet, which was most commonly the utilization of supplemental nutrition.

A majority of parents also reported that their child avoided, was attracted to, or was afraid of certain sounds. Forty-six percent (46%) reported that their child avoided certain sounds, 46% reported that their child was attracted to certain sounds, and nearly 54% reported that their child was afraid of certain sounds. In total, 62% of parents reported that their child avoided, was attracted to, or was afraid of certain sounds.

**Table 1** displays the mean scores for language, cognition, and motor abilities on the Bayley-III. These results demonstrate delays in all areas, with children with WS scoring greater than one standard deviation below the mean in each domain.

**Table 1:** Mean Scores on the Bayley-III.

	*N*	Mean	Standard deviation
Cognitive Composite	20	71.4	13.7
Language Composite	12	74.3	10.8
Receptive Communication	12	6.3	2.4
Expressive Communication	12	5.2	1.9
Motor Composite	8	65.5	7.4
Fine Motor	10	4.8	1.8
Gross Motor	8	3.9	1.7


A repeated measures analysis of variance was conducted to determine the difference in means between the cognition, language, and motor domains on the Bayley-III. A significant difference was found [*F*(2,5) = 9.23, *p* = 0.02, $\eta_{p}^{2}$ = 0.79]; pairwise comparisons revealed that the average language score (*M* = 74.3) was significantly higher than the average motor score (*M* = 65.5). A paired-samples *t*-test was conducted to evaluate the difference between the Receptive and Expressive language domains on the Bayley-III. Receptive language (*M* = 6.3, *SD* = 2.4) was significantly stronger than Expressive language [*M* = 5.2, *SD* = 1.9; *t*(11) = 2.38, *p* = 0.036, Cohen’s *d* = 0.69]. No significant difference was found between the Gross Motor and Fine Motor subtest scores.

### Adaptive Behavior

**Table 2** displays the mean scores for the measure of adaptive behavior (ABAS-2). Again, delays were observed in all areas, with the overall level of adaptive behavior appearing within the Mildly Delayed range (*M* = 64.0, *SD* = 10.6).

**Table 2:** Mean Scores on the ABAS-2.

	*N*	Mean	Standard deviation
General Adaptive Composite	15	64.0	10.6
Conceptual Domain	15	68.5	12.5
Communication	15	5.1	2.5
Functional Pre-Academics	11	5.1	2.6
Self-Direction	15	5.2	2.7
Social Domain	15	71.9	11.8
Leisure	15	5.6	2.8
Social	15	5.5	2.4
Practical Domain	15	67.9	11.6
Community Use	11	5.2	1.7
Home Living	11	5.0	2.6
Health and Safety	15	5.5	2.8
Self-Care	15	3.7	2.0


**FIGURE 1:**
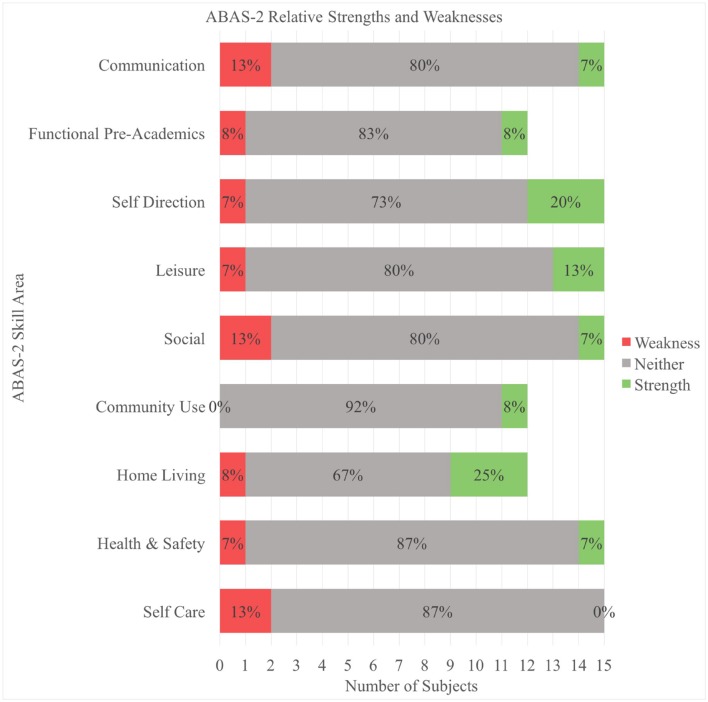
**Graph displaying percentages of participants that displayed a relative strength, weakness, or neither across skill sets.** Home Living, Functional Pre-Academics, and Community Use skill sets do not include participants under the age of 1 year.

Relative strengths and weaknesses for each individual were calculated based on a protocol provided in the handbook for the ABAS-2. An individual’s relative strength or weakness for each skill area was calculated by determining if the skill area score was significantly higher or lower relative to the individual’s other skill areas scores within that domain. **Figure 1** displays the relative strengths and weaknesses within each skill area for this sample. Heterogeneity was found, with no single area appearing as an overall strength or weakness. A repeated measures ANOVA between the three ABAS-2 adaptive behavior domains (Conceptual, Social, and Practical) indicated no significant difference between the three domains. A repeated measures ANOVA was also utilized to compare scores on the skill areas within each of the three ABAS-2 adaptive behavior domains. No significant difference was found between the skill areas within the Conceptual or Social domains, but a significant main effect was noted between the skill areas within the Practical domain (Home Living, Community Use, Health and Safety, Self-Care) [*F*(3,8) = 9.67, *p* = 0.005, $\eta_{p}^{2}$ = 0.78]. An examination of pairwise comparisons indicated that scores in Community Use were significantly higher than scores in Self-Care (*p* = 0.006).

Using the handbook provided by the authors of the ABAS-2, a test age was computed for each child for each of the skill areas. For each skill area, the child’s test age was then correlated with their chronological age. A significant positive correlation was found between mental age and chronological age within each skill area. The strongest correlations came from the Communication and Community Use areas, with the correlations being *r* = 0.942, and *r* = 0.949, respectively. There was a negative correlation (–0.499, *p* = 0.049) between the ABAS General Composite standard score and chronological age, suggesting that the gap between typically developing children and those with WS is widening between the ages of infancy and five years.

## Discussion

The aim of this study was to characterize the overall cognitive, developmental, and adaptive behavior characteristics of infants and toddlers with WS. This is the first study to utilize the ABAS-2 in the characterization of adaptive behavior in WS, and the findings point to skill areas that can be targeted for intervention.

### Developmental/Cognitive

Fewer parents than expected reported problems with eating and sleeping in their child, as well as their child avoiding, being afraid of, or being attracted to certain sounds. Previous studies have found up to 97% of parents reporting that their child has difficulties falling or staying asleep, 71% experiencing feeding difficulties, and 84% of parents reporting that their child had mild-moderate hyperacusis (Morris et al., 1988; Annaz et al., 2011). However, the relatively low number of parents reporting that their child avoided, was afraid of, or was attracted to certain sounds could be due to the fact that the current sample included four infants under the age of 1 year, and it is difficult for parents to determine if their infant is avoiding or attracted to certain sounds at this age. Also, as Annaz et al. (2011) utilized an older sample in their study, the sleep differences could be due to the fact that sleep problems become more prevalent as the child ages. This claim is further supported by the findings of Axelsson et al. (2013), who found evidence of sleep disturbance in infants and toddlers with WS, but not to the degree found by Annaz et al. (2011) Further research is needed to investigate prevalence of sleeping, eating, and hyperacusis in WS, as these issues are extremely difficult for both the child and his/her family.

Scores on the Bayley-III were at least 1 standard deviation below the mean across all areas. These delays were expected, as mild-moderate cognitive delays are common in individuals with WS (Mervis and Klein-Tasman, 2000). Previous studies with WS have shown that receptive language is usually a relative strength in comparison to expressive language (Mervis et al., 2003; Brock, 2007), and this study obtained similar results. It was also expected that language skills would be significantly higher than motor skills, as reported by Mervis and Bertrand (1997). However, there is very little research using the Bayley Scales as a measure of cognitive development, most likely due to the limited research conducted within this age group. It is also important to note that the previous study did not use the Third Edition of the Bayley Scales. Previous editions of the Bayley did not include a language domain, and instead included communication as a component of the mental domain. Furthermore, the communication component was not further separated into expressive and receptive language. More research is needed measuring cognitive ability in infants and toddlers with WS using the Bayley Scales of Infant and Toddler Development in order to offer a more thorough comparison to our current findings.

### Adaptive Behavior

On the ABAS-2, deficits were noted across all domains in this young infant/toddler WS population, similar to the deficits found in older children with WS (Greer et al., 1997; Mervis et al., 2001; Hahn et al., 2014). However, it is important to note that all of the previous adaptive behavior research utilized the VABS. The current study is believed to be the first to use the ABAS-2, and further research is needed in order to achieve a more detailed comparison, as the VABS domains do not directly correspond with the ABAS-2 domains. In the area of strengths and weaknesses of adaptive behavior, no specific area was found to be an overall strength or weakness, similar to the heterogeneity previously reported (Brawn and Porter, 2014).

The current finding that Self-Care scores are significantly lower than Community Use scores verifies an area in need of future research. Additional research is needed to discern if these results can be replicated in a larger sample, with further investigation into the cause of the low Self-Care scores. It is possible that the scores are significantly lower on this skill set, as the first eight questions relate to sleeping and eating behaviors, which are issues common to infants and toddlers with WS. It is also a possibility that in the young infant/toddler population, parents are choosing to do more for their child, which is causing some of these skills to develop at a slower rate.

The significant positive correlation found between each individual’s test age and chronological age on the ABAS-2 is similar to what has been found in previous studies in 4 to 8 year-old children (Mervis et al., 2001). In the population of infants and toddlers with WS, adaptive skills are increasing significantly as a function of chronological age, with no skills hitting a ‘plateau’ of no improvement. The negative correlation between the ABAS-2 Composite standard score and chronological age suggests that the gap between infants and toddlers with WS and infants and toddlers with typical development continues to grow between the ages of infancy and 5 years. This finding is not surprising, as few demands are placed on infants. However, as infants grow older, more is expected of them and this is where delays become more evident. However, this decline does not seem to continue as the child ages, as previous research with four to 8-year-old children found no association between standard score and chronological age on the VABS (Mervis et al., 2001). Future research should investigate this relationship using the ABAS-2 with older children.

### Limitations

There are limitations within the present study. The use of retrospective data limited the sample size, as some potential subjects did not have complete data sets. Results of this study should be considered preliminary, and interpreted with caution, due to the small sample size. Also, much of the data used in this study, including the intake form and ABAS-2, comes from parent report. Therefore, it is possible that parents may have overestimated or underestimated aspects of their child’s development and behavior, based on their perceptions of their child at that time. However, this study offers important and novel information regarding the development and adaptive behavior profiles of infants and toddlers with WS.

## Conclusion

The findings in the current study are similar to previous studies concerning children with WS, suggesting that the delays displayed in children with WS begin as early as infancy. Further research is needed to create a more robust profile of infants and toddlers with WS. Additional research utilizing the Bayley-III and the ABAS-2 is needed as well, as previous editions of the Bayley did not include a separate language domain, and the ABAS-2 domains have been shown to better align with the AAIDD definition of adaptive behavior. Further research is needed in order to determine potential reasons underlying the low scores in Self-Care, and future interventions that may help with this skill. Specifically, interventions targeting sleep and feeding difficulties should be investigated, as these skills may be contributing to the low Self-Care scores. Many children in this study were reported as being on a special diet; however, more research is needed to investigate the effect these feeding interventions have on self-care skills. Along with this, more research is needed to more fully characterize their sleeping and eating problems, as well as their underlying causes, so that interventions may be specifically designed for children with WS. The current study highlights the need for early intervention in these young children across all areas of development, particularly in self-care skills.

## Author Contributions

MM and RK assisted with the design of the study, data entry, data analysis, and manuscript preparation. RA assisted with data formatting and created template for data entry.

## Conflict of Interest Statement

The authors declare that the research was conducted in the absence of any commercial or financial relationships that could be construed as a potential conflict of interest.
