# Supplementary material for: Adaptive Behavior and Development of Infants and Toddlers with Williams Syndrome
Source: Front Psychol. 2016 Apr 28;7:598. doi: 10.3389/fpsyg.2016.00598 (PMC4848290; doi:10.3389/fpsyg.2016.00598)
Supplement: Supplementary file 1 [file Data_Sheet_1.DOC]

***Supplementary Material***

Adaptive Behavior and Development of Infants and Toddlers with Williams Syndrome

Rebecca Kirchner^1,2^BS, Marilee Martens^3,4^*PhD, and Rebecca Andridge^5^ PhD

^1^The Ohio State University, Department of Neuroscience, Columbus, Ohio, USA

^2^The Ohio State University, Department of Psychology, Columbus, Ohio, USA

^3^The Ohio State University at Newark, Department of Psychology, Newark, Ohio, USA

^4^The Ohio State University Nisonger Center, Columbus, Ohio, USA

^5^The Ohio State University, Department of Biostatistics, Columbus, Ohio, USA

*** Correspondence:** Marilee Martens, The Ohio State University at Newark, Department of Psychology 1179 University Drive, Newark, Ohio, 43055

martens.22@osu.edu

**Williams Syndrome Clinic**

**INTAKE FORM**

**Health and Development History**

| Date | | | | |
| --- | --- | --- | --- | --- |
| Child’s Full Name | | | | Date of Birth |
| Your Full Name(s) | | | | Relationship to Child |
| Telephone (Home) | | | | (Work) |
| Email Address | | | | (Cell) |
| Home Address | | | | |
| Name of School | | | | Grade |
| School Address | | | | |
|  | School IEP Team – Name and Title |  |  | |
|  |  |  |  | |
|  |  |  |  | |
|  |  |  |  | |

| Primary Care Provider (i.e. pediatrician, family practitioner, nurse practitioner): |  |
| --- | --- |
| In addition to yourself, to whom would you like the report sent? (primary care provider) Please include the address. | |
|  |  |
|  |  |

Please list the question(s) you would like answered once the evaluation is completed:

| 1. | |
| --- | --- |
| 2. | |
| 3. | |
| 4. | |
| Whose idea was it that your child have an evaluation? |  |

**Birth History**

Which of the mother’s pregnancies was this? (1^st^, 2^nd^, etc.)? ________________________________

Has the mother had miscarriages?  Yes  No Previous premature baby(ies)? ______________________________________

***During pregnancy*** (please check Yes or No if the following occurred. If Yes, please describe briefly)

| Yes  No | Illness/infection/accident | Describe: ______________________________________________________________ |
| --- | --- | --- |
| Yes  No | Medication taken | Describe: ______________________________________________________________ |
| Yes  No | Smoking | If yes, how much: ______________________________________________________________ |
| Yes  No | Alcohol intake | If yes, how much: ______________________________________________________________ |
| Yes  No | Drug intake | If yes, how much: ______________________________________________________________ |
| Weight gain in pounds (during pregnancy): ________________________________________________________________________ | | |
| Length of pregnancy in weeks (most babies are born between 38-42 weeks): ______________________________________________ | | |

***Labor***

| Yes  No | Induced |
| --- | --- |
| Yes  No | Lasted over 12 hours |

***Delivery***

| Yes  No | Caesarean section |  |
| --- | --- | --- |
| Yes  No | Anesthesia | If yes, what type:  Spinal  Epidural  General (asleep) |
| Yes  No | Complications | If yes, describe _________________________________________________ |

***Newborn***

| Birth weight: _______________________________ Cried right away:  Yes  No | | |
| --- | --- | --- |
| Apgar scores, if known: ________________ 1 minute ______________________ 5 minutes | | |
| Yes  No | Complications | If yes, describe: ______________________________________________________________ |
| Yes  No | Breast fed | If yes, how long?: ______________________________________________________________ |
| Went home after ________ days in the hospital | | |

***Infancy***

| Yes  No | Enjoyed cuddling |
| --- | --- |
| Yes  No | Fussy, irritable Comments: ________________________________________________________ |
| Yes  No | More active than others ________________________________________________________ |
|  | |

***Diagnosis***

| Age at which child was diagnosed with Williams Syndrome ___________________________________________________________ | |
| --- | --- |
|  | FISH (fluorescence *in situ* hybridization) test used to confirm?  Yes  No |
| Who did FISH testing? _________________________________________________________________________________________ | |

**History of Infancy, Childhood and Adolescence**

| A. Immunization record: | Up to date  Some immunizations not given. Please explain:_________________________________________ | | | | | | | | | | |
| --- | --- | --- | --- | --- | --- | --- | --- | --- | --- | --- | --- |
|  | ___________________________________________________________________________________ | | | | | | | | | | |
| B. Childhood diseases (age or N/A): | | | | | | | | | | | |
| Mumps ______________________________________ | | | | | |  | | Measles _______________________________________ | | | |
| German Measles ______________________________ | | | | | |  |  | Chicken Pox ___________________________________ | | | |
| Roseola _____________________________________ | | | | | |  |  | Whooping Cough _______________________________ | | | |
| Scarlet Fever _________________________________ | | | | | |  |  | Meningitis/Encephalitis __________________________ | | | |
| C. Check other problems child has now or has had in the past: | | | | | | | | | | | |
|  | | | Now | | Past |  | |  | | Now | Past |
| Recurrent stomach aches | | | _____ | | _____ |  |  | Pneumonia or Bronchitis | | _____ | ____ |
| Urine problems | | | _____ | | _____ |  |  | Persistent vomiting | | _____ | ____ |
| Frequent constipation | | | _____ | | _____ |  |  | Persistent diarrhea | | _____ | ____ |
| Seizures | | | _____ | | _____ |  |  | Headaches | | _____ | ____ |
| Recurrent infections | | | _____ | | _____ |  |  | Dizziness | | _____ | ____ |
| Ear infections | | | _____ | | _____ |  |  | Unconsciousness | | _____ | ____ |
| Accidents (head injuries, other injuries, broken bones?) | | | _____ | | _____ |  |  | Asthma | | _____ | ____ |
| Unusual movements | | | _____ | | _____ |  |  | Tics | | _____ | ____ |
|  |  |  |  |  |  |  |  |  |  |  |  |
| C. If you checked any of the previous problems, please describe further: | | | | | | | | | | | |
| Other problems not included in the above list (please specify) __________________________________________________________ | | | | | | | | | | | |
| Do you have any concerns about your child’s size or growth?  Yes  No If *yes,* please describe:   |  | | --- |   Does your child have a heart abnormality?  Yes  No If *yes,* please describe:   |  | | --- |   Does your child have any other physical disability or impairment?  Yes  No If *yes,* please describe:   |  | | --- | |  | | | | | | | | | | | | |
| If female and past puberty, age when period (menstruation) began ______________________________________________________ | | | | | | | | | | | |
| Do you have any concerns about your child’s sexual development? _____________________________________________________ | | | | | | | | | | | |
| D. Does your child have any known allergies or sensitivities to food or drugs?  Yes  No  Do not know | | | | | | | | | | | |
| If *yes*, please explain: __________________________________________________________________________________________ | | | | | | | | | | | |
| ____________________________________________________________________________________________________________ | | | | | | | | | | | |
| E. Does your child have any problems associated with going to bed or sleeping?  Yes  No  Do not know | | | | | | | | | | | |
| If *yes*, please explain: __________________________________________________________________________________________ | | | | | | | | | | | |
| ____________________________________________________________________________________________________________ | | | | | | | | | | | |
| ____________________________________________________________________________________________________________ | | | | | | | | | | | |
| Is your child currently taking any medication or has he/she received prescribed medication (for a month or longer) in the past? | | | | | | | | | | | |
| Yes  No | | | | | | | | | | | |
| If *yes*, please answer the following: | | | | | | | | | | | |
| Medication | | Amount taken per day | | Is the child still taking medication? | | | | | Why is/was it taken? | | |
|  |  |  |  | Yes | | | No | |  |  |  |
|  | |  | |  | | |  | |  | | |
|  | |  | |  | | |  | |  | | |
|  | |  | |  | | |  | |  | | |
|  | |  | |  | | |  | |  | | |
|  | |  | |  | | |  | |  | | |
|  | |  | |  | | |  | |  | | |
|  | |  | |  | | |  | |  | | |

17. Is there a history on either side of the child’s family of conditions such as birth defects, diabetes, seizures, mental retardation, mental or emotional disorders, childhood hearing loss, or learning disabilities?

Yes  No  Do not know

| If *yes*, please explain: |  |
| --- | --- |
|  | |
|  | |

**Growth and Development**

| Does your child have any feeding difficulties?  Yes  No If *yes*, please explain: |
| --- |
|  |
|  |
|  |
|  |

List any foods that are avoided:

|  |
| --- |
|  |
|  |

Does your child receive any special foods, formulas, vitamins, dietary supplements, or is he/she on a special diet?

Yes  No If *yes*, please explain:

|  |
| --- |
|  |
|  |

Has your child’s vision ever been tested?

Yes  No  Do not know

If *yes*, what were the results? If no, do you have concerns?

|  |
| --- |
|  |

Who conducted the testing?

Name_______________________________________________________________________________________________________

When was the testing done? _____________________________________________________________________________________

Has your child’s hearing ever been tested?

Yes  No  Do not know

If *yes*, what were the results? If no, do you have concerns?

|  |
| --- |
|  |

Who conducted the testing?

Name_______________________________________________________________________________________________________

When was the testing done? _____________________________________________________________________________________

**Hyperacousis**

1. Does your child avoid certain sounds?  Yes  No

2. Is your child attracted to certain sounds?  Yes  No

3. Is your child afraid of certain sounds?  Yes  No

Explain:

|  |
| --- |
|  |
|  |

**Developmental History**

|  | **Age** | **Not Yet Developed** |
| --- | --- | --- |
| Sat without support |  |  |
| Crawled |  |  |
| Stood without support |  |  |
| Walked without assistance |  |  |
| Threw ball |  |  |
| Spoke first words |  |  |
| Said phrases |  |  |
| Said sentences |  |  |
| Showed clear hand preference |  |  |
| Bowel trained |  |  |
| Bladder trained, day |  |  |
| Bladder trained, night |  |  |
| Rode tricycle |  |  |
| Rode bicycle (without training wheels) |  |  |
| Tied shoelaces |  |  |
| Named colors |  |  |
| Named coins |  |  |

**SIBLINGS**

| **Name** | **Age** | **Any medical, social, academic, speech problems?** |
| --- | --- | --- |
|  |  |  |
|  |  |  |
|  |  |  |
|  |  |  |
|  |  |  |

**Play / Leisure Time**

How and with whom does your child like to spend his/her play or leisure time?

|  |
| --- |
|  |
|  |

How much supervision does your child need during play time?

Total Supervision  Some Supervision

A great deal of supervision  Almost no supervision

**Family Life**

Are there family circumstances that seem to have had a positive effect on your child’s development, such as support from relatives, outside activities, etc.?

Yes  No  Do not know

If *yes*, please explain:

|  |
| --- |
|  |

Activity, Attention, Behavior

| *Please check appropriate column* | **Not True** | **Sometimes True** | **Very/Often True** |
| --- | --- | --- | --- |
| 1. Fails to finish things he/she starts |  |  |  |
| 2. Can’t concentrate, can’t pay attention for long |  |  |  |
| 3. Can’t sit still, restless, or hyperactive |  |  |  |
| 4. Fidgets |  |  |  |
| 5. Daydreams or gets lost in his/her thoughts |  |  |  |
| 6. Impulsive or acts without thinking |  |  |  |
| 7. Has difficulty following directions |  |  |  |
| 8. Talks out of turn |  |  |  |
| 9. Messy work |  |  |  |
| 10. Is inattentive, is easily distracted |  |  |  |
| 11. Talks too much |  |  |  |
| 12. Fails to carry out assigned tasks |  |  |  |
| 13. Disturbs other children |  |  |  |
| 14. Demands must be met immediately |  |  |  |
| *Please check appropriate column* | **Not True** | **Sometimes True** | **Very/Often True** |
| 15. Is easily frustrated |  |  |  |
| 16. Cries often and easily |  |  |  |
| 17. Mood changes quickly and drastically |  |  |  |
| 18. Has temper outbursts, is explosive |  |  |  |
| 19. Head bangs |  |  |  |
| 20. Rocks in bed |  |  |  |
| 21. Is self-destructive |  |  |  |
| 22. Is difficult to comfort |  |  |  |
| 23. Is stiff/rigid |  |  |  |
| 24. Exhibits looseness/floppiness |  |  |  |
| 25. Is shy with strangers |  |  |  |
| 26. Is shy with peers |  |  |  |
| 27. Shows extreme reaction to noise |  |  |  |
| 28. Has difficulty keeping to schedule or schedule changes |  |  |  |
| 29. Has difficulty getting satisfied |  |  |  |
| 30. Fails to be affectionate towards parents |  |  |  |
| 31. Is cruel to animals |  |  |  |
| 32. Tics (excessive movements/odd sounds) |  |  |  |

Describe your child’s personality – moods, relationships, behavior:

____________________________________________________________________________________________________________

____________________________________________________________________________________________________________

____________________________________________________________________________________________________________

Describe your child’s strengths:

|  |
| --- |
|  |
|  |
|  |
|  |

Please add any additional information which you feel may help us better understand your child:

|  |
| --- |
|  |
|  |

# Supplementary Figure 1. Nationwide Children’s Hospital/Nisonger Center Williams Syndrome Clinic Intake Form.
